# Assessment of the prevalence of respiratory pathogens and the level of immunity to respiratory viruses in soldiers and civilian military employees in Poland

**DOI:** 10.1186/s12931-025-03142-8

**Published:** 2025-02-21

**Authors:** Aleksandra Nakonieczna, Magdalena Kwiatek, Karolina Abramowicz, Magdalena Zawadzka, Izabela Bany, Patrycja Głowacka, Katarzyna Skuza, Tomasz Lepionka, Paweł Szymański

**Affiliations:** 1 Military Institute of Hygiene and Epidemiology, Biological Threats Identification and Countermeasure Center, 24-100 Puławy, Poland; 2 Military Institute of Hygiene and Epidemiology, 01-163 Warsaw, Poland; 3 Department of Epidemiology and Public Health, Medical University of Łódź, 90-419 Łódź, Poland; 4 Department of Pharmaceutical Chemistry, Drug Analyses and Radiopharmacy, Medical University of Łódź, 90-151 Łódź, Poland

**Keywords:** Respiratory pathogens, Vaccines, Immunity, Influenza, COVID-19, Military personnel, Antibodies

## Abstract

**Background:**

This study provides a detailed analysis of respiratory tract infections (RTIs) and immunity levels against influenza and SARS-CoV-2 among soldiers and military personnel in Poland. Owing to their unique service environments, this occupational group is at high risk. During deployments, they often face adverse physical conditions, close living quarters, and exposure to both local and endemic pathogens. It particularly increases their susceptibility to RTIs, which remain a leading cause of illness worldwide.

**Methods:**

The study cohort included 379 participants aged between 19 and 60 years. We used polymerase chain reaction (PCR) techniques to detect 34 common respiratory pathogens and analyzed blood serum samples to assess the degree of immunity against the influenza A, B, and SARS-CoV-2 viruses. In 78.10% of the participants, at least one respiratory pathogen was detected.

**Results:**

Human rhinovirus (HRV) was the most common (8.71%), followed by SARS-CoV-2 (4.75%) and influenza A (H1N1) sw (2.90%). *Staphylococcus aureus* was the most prevalent bacterial pathogen (18.47%), with significant occurrences of *Haemophilus influenzae* (14.24%) and *Klebsiella pneumoniae* (9.76%). Additionally, 52.3% of those with coinfections had combinations of bacterial and viral pathogens, highlighting the complexity of diagnosing and managing these infections. We also assessed immunity levels, which focused on antibodies specific to influenza A/B and SARS-CoV-2 viruses. For all the results obtained, statistical analyses were performed. A weak positive correlation between age and levels of anti-influenza antibodies was observed, suggesting a slight increase in antibody levels with age. A total of 81.53% of the participants had received at least one dose of the SARS-CoV-2 vaccine. A significant correlation between the number of vaccine doses and higher anti-SARS-CoV-2 IgG antibodies was observed, indicating stronger immunity with more vaccinations.

**Conclusions:**

This study underscores the importance of specialized health monitoring and preventive measures such as vaccinations to protect military personnel from RTIs and maintain their operational readiness. The detailed analysis of pathogen prevalence and immunity levels offers valuable insights into this occupational group's health risks and needs.

*Clinical trial number*: Not applicable.

## Background

Respiratory tract infections (RTIs) are a significant challenge to medicine worldwide, as they can lead to severe complications and even death. They are among the leading causes of visits to primary healthcare centers [1]. For some occupational groups, the risk of acquiring RTIs is greater. For example, it is common for professional soldiers to be exposed to adverse environmental conditions during their duties, including dangerous physical or biological factors [2]. A factor predisposing individuals to the spread of infectious diseases is being in areas with lowered hygiene standards. This applies to exercises carried out on training grounds and deployments outside the country as part of the Polish Military Contingents, where local endemic diseases, uncommon in Poland, occur. Additionally, specific climates, dust, emissions from burn pits, and industrial pollutants contribute to the rapid spread of diseases. Soldiers in moderate climate conditions, often clustered together during military exercise, are particularly exposed to the transmission of infectious diseases, including seasonally occurring pathogens of the upper respiratory tract [3]. Infections caused by so-called respiratory pathogens include upper and lower RTIs. Upper respiratory tract infections (URTIs) affect the nose, throat, sinus, and larynx, and in 80–90% of cases, they are caused by viruses. Among them, human rhinoviruses (HRVs) A/B/C, human adenoviruses (HAdVs) B/C/E, human respiratory syncytial virus (RSV), influenza A virus (IAV), influenza B virus (IBV), and human coronaviruses (SARS-CoV-2, 229E, NL63, OC43, and HKU1) are the most common [4, 5]. In typical colds, systemic symptoms are rarely observed. The appearance of these viruses is usually associated with infections caused by influenza, parainfluenza (PIV), or adenoviruses. Lower respiratory tract infections (LRTIs) affect the trachea, bronchi, bronchioles, or lungs and have a mixed, viral‒bacterial etiology [6]. Influenza and parainfluenza viruses, adenoviruses, and coronaviruses are all commonly responsible for LRTIs [5].

The Department of Infectious Disease Epidemiology and Surveillance of the National Institute of Public Health-National Institute of Hygiene (NIPH-NIH; https://www.pzh.gov.pl/) regularly prepares and publishes epidemiological reports covering infectious diseases in Poland. According to reports published by the NIPH-NIH for 2023, the influenza virus is behind the highest number of RTIs, with a total of 3 781 241 cases, including suspected cases. SARS-CoV-2 was the second most common virus, with 380,753 cases, and HRSV was the third, with 12,054 cases. The latter, however, had the highest rate of hospitalized patients (33.1%). As reported, invasive bacterial RTIs are less common, but they are significantly more dangerous, as they tend to be responsible for a much higher percentage of hospitalizations. Based on the data available for the same year, three most common RTI bacteria were *Streptococcus pyogenes, Streptococcus pneumoniae,* and *Bordetella pertussis* causing invasive diseases in 44,734, 2960, and 927 patients, respectively, with *S. pneumoniae* infections having a hospitalization rate of 98.7% ([7], EPIMELD, 2024; https://wwwold.pzh.gov.pl/oldpage/epimeld/2023/Ch_2023.pdf). Owing to the high morbidity rate of influenza (flu), emerging pandemics, and significant mortality rate (approximately 500,000 deaths per year worldwide [8]), many prophylactic vaccinations have been developed, which are currently considered the primary preventive measures for this disease. An appropriate selection of the most current circulating strains may increase the effectiveness of the vaccines by up to 60%, whereas the content of less recent strains in the vaccine reduces its activity to 10–20%. Interestingly, influenza vaccines increase the levels of antibodies (anti-hemagglutinin-specific IgG) but not as effectively as natural viral infection does [9]. Similar to influenza, COVID-19 vaccination has been implemented to reduce the risk of dangerous complications and deaths and prevent the recurrence of the pandemic. The effectiveness of the vaccine, i.e., preventing the onset of any clinical symptoms, ranges from 40 to 60% after a single dose but increases to 85% in fully vaccinated individuals (after two doses). The level of IgG against the spike protein of SARS-CoV-2 is effectively increased by vaccination and maintained for at least six months [10].

Our article presents the results of an extensive health status analysis in the context of respiratory infections conducted under the auspices of the Ministry of Defense among 379 soldiers and civilian military employees in Poland. This project aimed to study the prevalence of the 34 most common respiratory pathogens and assess participants' immunity to influenza and SARS-CoV-2 viruses by quantifying specific antibodies in their blood serum samples.

## Materials and methods

### Participants

The study cohort included 379 participants, primarily soldiers. The surveyed volunteers came from several military units, such as, e.g. the road and bridge battalion, EOD regiment, armament depot, territorial defense brigade, Polish Air Force Academy, and Engineering and Aviation Training Center. Soldiers from these units frequently serve in various outdoor environments, such as open spaces and river areas, as well as warehouses or hangars, and they hold training ground exercises. Moreover, some of them are also involved in border security measures and responding to emergencies like floods. For these reasons, they may be more vulnerable to RTI infections.

Our research was conducted following the World Medical Association's Declaration of Helsinki and was approved by the Bioethics Committee of the Military Medical Board in Warsaw (Resolution No. 11/23). The participants were informed about the details of the study, its purpose, and the benefits of their participation. All the volunteers provided written informed consent as a condition to participate in the study and completed a original survey regarding their current health conditions and passed infections and vaccinations before sampling.

### Sampling

Naso- and oropharyngeal swabs and blood samples were collected from 379 soldiers and civilian military personnel in Polish military units during the period of the highest seasonal increase in respiratory diseases, i.e., between October 2023 and February 2024. The samples were collected by qualified medical personnel authorized to collect and transport biological material. The collected blood samples and swabs were placed in tubes with virus transport and preservation medium (inactivated) from BioComma (Guangdong, China), stored at 2–8 °C and shipped to the laboratory.

### Tests for the presence of respiratory pathogens

This study employed two different systems with different kits. Manually isolated genetic material was used to perform real-time PCR on a CFX96 Real-Time PCR instrument (Bio-Rad, Hercules, CA, USA), while the Roche Cobas 6800 system (Roche Diagnostics Corporation, Indianapolis, IN, USA) was used for automatic PCR detection of the SARS-CoV-2 virus.

*FTD*: Genetic material extraction was performed from an initial volume of 200 µl using a QIAamp DNA kit (Qiagen, Valencia, CA, USA) according to the manufacturer's instructions, followed by elution in 100 µl of elution buffer. During extraction, 3.6 µl (per sample) of internal control (IE) was added to the lysis buffer solution. A Fast Track Diagnostics® (FTD) Respiratory Pathogens 33 (RUO) Kit (Siemens Health engineers, Erlangen, Germany) based on the real-time RT‒PCR (reverse-transcriptase polymerase chain reaction) method was utilized for the detection of respiratory viruses, bacteria, and fungi in the extracts. The tested microorganisms are listed in Table 1.

**Table 1:** List of respiratory pathogens detected by the FTD Respiratory Pathogens 33 (RUO) Kit

Viruses	Influenza A, B, and C, influenza A (H1N1) (swine lineage), parainfluenza viruses 1–4, human coronaviruses (HCoV: NL63, 229E, OC43, and HKU1), human metapneumoviruses A and B, human respiratory syncytial viruses A and B, rhinovirus, adenovirus, bocavirus, enterovirus, parechovirus
Bacteria	*Chlamydia pneumoniae*, *Mycoplasma pneumoniae*, *Streptococcus pneumoniae*, *Haemophilus influenzae*, *Haemophilus influenzae* type B, *Staphylococcus aureus*, *Moraxella catarrhalis*, *Bordetella* spp. (excluding *B. parapertussis*), *Klebsiella pneumoniae*, *Legionella pneumophila*/*Legionella longbeachae*, *Salmonella* spp.
Fungi	*Pneumocystis jirovecii*

*COBAS*: Sample examination was conducted via the Cobas SARS-CoV-2 qualitative assay for the Cobas 6800/8800 systems (Roche Molecular Systems), which allows the detection of two genes: the E gene, which is common to all members of the Sarbecovirus subgenus family, which includes the SARS-CoV-2 virus, and a SARS-CoV-2-specific gene, ORF1ab. This assay relies on the automatic extraction and purification of nucleic acids from 400 µl samples, followed by PCR.

### Evaluation of the degree of immunity against influenza and SARS-CoV-2 viruses

The humoral and cellular immune responses were examined via enzyme-linked immunosorbent assay (ELISA) in blood serum samples to assess the participants' immunity to previous contact/infection with a virus or vaccination comprehensively.

*Humoral response*: Full blood samples were taken into BD Vacutainer® SST II Advance tubes with a clot activator and separating gel (BD, Franklin Lakes, NJ, USA) to obtain blood serum. The material was then used to detect antibodies against influenza A and B and SARS-CoV-2 viruses via ELISA. The tests were performed with the use of commercially available In Vitro Diagnostics (IVD) tests for the quantitative detection of IgG by EUROIMMUN (Wrocław, Poland), i.e., Anti-virus influenza A ELISA (IgG) (Ref: EI 2691–9601 G), Anti-virus influenza B ELISA (IgG) (Ref: EI 2692–9601 G), and Anti-SARS-CoV-2 QuantiVac ELISA (IgG) (Ref: EI 2606–9601-10 G). ELISA microtiter plates for the respective virus-specific antibodies were coated with the antigens derived from the following isolates: the “Texas” H3N2, "Singapore" H1N1, and “California” H1N1sw isolates for the IAV test; the “Hongkong 5/72” isolate for the IBV test; and the S1 domain from the Wuhan-Hu-1 isolate for the SARS-CoV-2 test. All tests were carried out according to the manufacturer's guidelines.

*Cellular response*: BD Vacutainer® Heparin Tubes were used to collect fresh whole blood. A commercially available interferon-gamma release assay (IGRA) (EUROIMMUN) was performed to evaluate the T-cell-mediated response. The principle of the test relies on in vitro stimulation of T cells with specific peptides of the spike antigen and then the quantification of the released interferon-gamma (IFN-γ). Briefly, 500 µl of blood was stimulated overnight (20–24 h) in 5% CO_2_ at 37 °C in the following tubes: BLANK (negative control), STIM (positive control), and TUBE (antigens based on the SARS-CoV-2 Wuhan Spike protein) from the Quan-T-Cell SARS-CoV-2 Kit (Ref: ET 2606–3003). Following incubation and centrifugation, the plasma was collected for IFN-γ analysis via the Quan-T-Cell-ELISA Kit (Ref: EQ 6841–9601).

### Software and statistical analysis

The collected material was analyzed statistically using Excel tools and Statistica 13.3 software. Descriptive statistics and parametric and non-parametric methods of data analysis were applied. The assessment of normality distribution was performed using the Shapiro‒Wilk test. In the absence of a normal distribution, the Mann‒Whitney U test was used to assess the relationships between quantitative variables. The Kruskal‒Wallis H test was used to analyze more than two groups of ordinal variables. The relationships between two qualitative variables were assessed via Pearson's Chi^2^ test of independence. The correlation coefficient was calculated to express the correlation between two variables. The r-Pearson correlation coefficient was used to describe the correlation between quantitative variables, and in the absence of a normal distribution, Spearman's rank correlation coefficient was used. For all analyses, p ≤ 0.05 was taken as the significance level.

## Results

### Participants

83% of the surveyed participants were soldiers, while the remaining 17% were civilian military personnel. The participants were in the 19–60 years age range and the mean age and median age were $\overline{x}$  = 41.18 and *M* = 42.0, respectively. Males and females represented 65.17% (*n* = 247) and 34.83% (*n* = 132) of the tested group, respectively.

### Prevalence of RTI pathogens

Among all 379 tested participants, any viral, bacterial, or fungal pathogens were confirmed in 296 people (78.10%), and the detected pathogens are listed in Table 2.

**Table 2:** Respiratory pathogens detected during the studies, with the respective number and percentage relative to the total number of tested participants (n = 379)

	Viral pathogens	Cases	
		n	%
**1**	**Human rhinovirus (HRV)**	**33**	**8.71**
**2**	**SARS-CoV-2**	**18**	**4.75**
**3**	**Influenza A (H1N1) virus (swine lineage) (IAV(H1N1)swl)**	**11**	**2.90**
**4**	**Human coronavirus 229E (HCoV 229E)**	**10**	**2.64**
**5**	**Human adenovirus (HAdV)**	**7**	**1.85**
**6**	**Enterovirus (EV)**	**6**	**1.58**
7	Influenza C virus (ICV)	2	0.53
8	Human parainfluenza virus 3 (HPIV03)	3	0.79
9	Human coronavirus OC43 (HCoV OC43)	2	0.53
10	Influenza A virus (IAV)	1	0.26
11	Human coronavirus NL63 (HCoV NL63)	1	0.26
12	Human metapneumovirus A and B (HMPV A&B)	1	0.26
13	Human bocavirus (HBoV)	1	0.26

	Bacterial and fungal pathogens	Cases	
		n	%
**1**	***S. aureus***	**70**	**18.47**
**2**	***H. influenzae***	**54**	**14.24**
**3**	***K. pneumoniae***	**37**	**9.76**
**4**	***M. catarrhalis***	**14**	**3.69**
**5**	***S. pneumoniae***	**13**	**3.43**
**6**	***P. jirovecii*** (fungus)	**9**	**2.37**
7	*H. influenzae* type B	1	0.26
8	*L. pneumophila/L. longbeachae*	1	0.26
9	*Bordetella* spp.	1	0.26

Pathogens with the highest detection rates in the tested population are bolded

The relationship between the occurrence of cold symptoms and the presence of pathogens was also verified. Cold symptoms such as runny nose, headache, sore throat, cough, chills, sinusitis, elevated temperature, and muscle pain within the last two weeks before sampling were noted in 21.90% (*n* = 83) of the participants. Statistically significant relationships were detected only concerning symptoms and human rhinovirus (HRV) (χ^2^ = 11.7259, df = 1, p = 0.00061), enterovirus (EV) (χ^2^ = 13.4525, df = 1, p = 0.00024), *S. pneumoniae* (χ^2^ = 4.63000, df = 1, p = 0.03141), and *H. influenzae* (χ^2^ = 6.43251, df = 1, p = 0.01120).

### Coinfections

The correlations between the presence of cold symptoms or elevated temperatures within two weeks before testing and the presence of coinfections were analyzed. A coinfection in our study was considered when two or more pathogens were detected. Owing to the small number of cases in which the fungus *P. jirovecii* was detected, only bacteria and viruses were used for analysis. Among the 86 identified coinfections, 52.3% (n = 45) were mixed, 37.2% were bacterial–bacterial coinfections, and 10.4% were viral–viral coinfections. Among these 86 individuals, 29 reported one or more of the cold symptoms. A statistically significant relationship was noted only between the presence of symptoms and viral-viral coinfections (χ^2^ = 5.614038, df = 1, p = 0.01782).

### Evaluation of the immunity level against IAV/IBV and SARS-CoV-2 viruses

For antibodies against IAV/IBV, SARS-CoV-2, and IFN-γ, the sample results were classified as positive when they reached the following concentrations: ≥ 22 RU/ml, ≥ 11 RU/ml, and > 200 mlU/ml, respectively. Statistical data analysis for four measurements of IgG levels, anti-INF-γ, anti-SARS-CoV-2, anti-IAV, and anti-IBV, was performed for the studied population. The values of variables (*N*), means $\left( {\overline{x} } \right)$ , medians (*M*), minimum and maximum values, upper quartiles (*Q*_*3*_), lower quartiles (*Q*_*1*_), and standard deviations (σ) for antibody levels were determined and are shown in Table 3. The limits of detection for the interpretation of the results were provided by the manufacturer. In the case of anti-IAV and anti-IBV IgG, the minimal limits of detection were 1.0 RU/ml and 0.4 RU/ml, respectively, and the upper threshold of quantitation was 200 RU/ml. For these assays, the result values exceeding 200 RU/ml were defined as > 200 RU/ml, and this value is shown in the graphs as the maximal value. For anti-SARS-CoV-2 IgG, the lower detection limit was 1.2 RU/ml, and the upper threshold of the result quantitation was 120 RU/ml. Similarly, higher result values were defined as > 120 RU/ml, and this value is shown in the graphs as the maximum. The lower limit for detection of INF-γ IgG was 18.44 mlU/ml.

**Table 3:** Descriptive statistics for the levels of anti-INF-γ, anti-SARS-CoV-2, anti-IAV, and anti-IBV IgG antibodies

	N	$\overline{x}$	M	Min	Max	Q_1_	Q_3_	σ
IgG anti-INF-γ	263	637.35	475.34	0.00	3379.61	212.80	898.18	563.18
IgG anti-SARS-CoV-2	379	106.17	120.00	0.00	120.00	103.20	120.00	26.10
IgG anti-IAV	379	79.67	78.30	0.90	200.00	56.06	107.00	36.56
IgG anti-IBV	379	122.21	128.00	0.00	200.00	83.00	168.90	53.85

The correlation between anti-IAV and anti-IBV antibody levels was studied. An increase in one antibody type causes an increase in the other. The positive correlation was moderate (ρ = 0.3414; p < 0.0000). The same assay was performed for the correlation between anti-INF-γ IgG and anti-SARS-CoV-2 IgG levels, and similarly, the correlation was positive but weak (ρ = 0.2687; p = 0.00001).

### Influence of age on SARS-CoV-2- and IAV/IBV-specific antibody levels

**Fig. 1:**
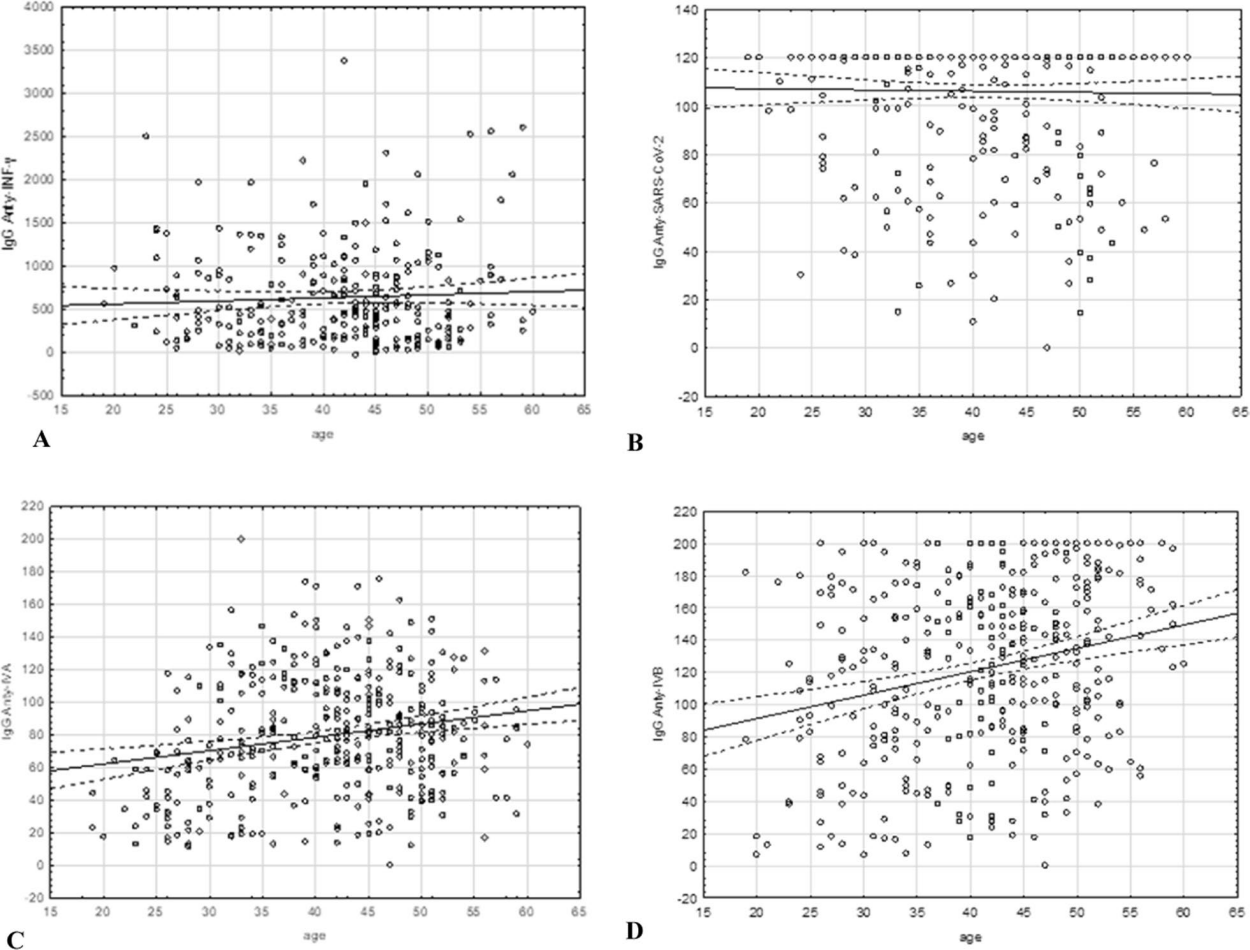
Dependence of particular IgG levels on the age of the participants: IgG anti-INF-γ (**A**), IgG anti-SARS-CoV-2 (**B**), IgG anti-IAV (**C**), and IgG anti-IBV (**D**). Anti-INF-γ IgG levels are given in mlU/ml, and all other antibody levels are given in RU/ml

Each IgG antibody level was analyzed for its relationship or correlation with the age of the participants. In the case of anti-INF-γ IgG and anti-SARS-CoV-2 IgG, no statistically significant relationship was found (p = 0.11145 for anti-INF-γ IgG and p = 0.61749 for anti-SARS-CoV-2 IgG) (Fig. 1a, b). Spearman's rank correlation coefficient revealed a statistically significant relationship between the level of anti-IAV (ρ = 0.172212; p = 0.00076) and anti-IBV IgG and age (ρ = 0.228514; p = 0.000007). In both cases, the antibody levels increased with age (Fig. 1c, d). The correlation value (ρ) for IgG anti-IAV and anti-IBV was weak (below 0.3).

### Influence of vaccinations and infections on antibody levels against COVID-19 and influenza

According to the surveys, seventeen participants had been diagnosed with flu during the past 12 months, 14 of whom were unvaccinated. Vaccines against influenza during this time were received by 9.50% (n = 36) of the participants. In the case of SARS-CoV-2, 81.53% (n = 309) of the participants were vaccinated. The number of doses of the SARS-CoV-2 vaccine ranged from one to four, and the percentages of nonvaccinated people (zero doses) those immunized with different numbers of doses were as follows: zero doses: 18.47% (n = 70); one dose: 19.26% (n = 73); two doses: 34.04% (n = 129); three doses: 26.91% (n = 102); and four doses: 1.32% (n = 5). Sixteen people confirmed COVID-19 infection in the past 12 months, and only one of them was unvaccinated. Therefore, very few people in our study group were unvaccinated and had been diagnosed within the last 12 months (14 people with flu and one with COVID-19). Therefore, in our study, we could not clearly assess the influence of each of these factors separately, especially the impact of past infection, and the outcomes we present show the levels of specific antibodies that may result from both vaccination and contact with the pathogen.

**Fig. 2:**
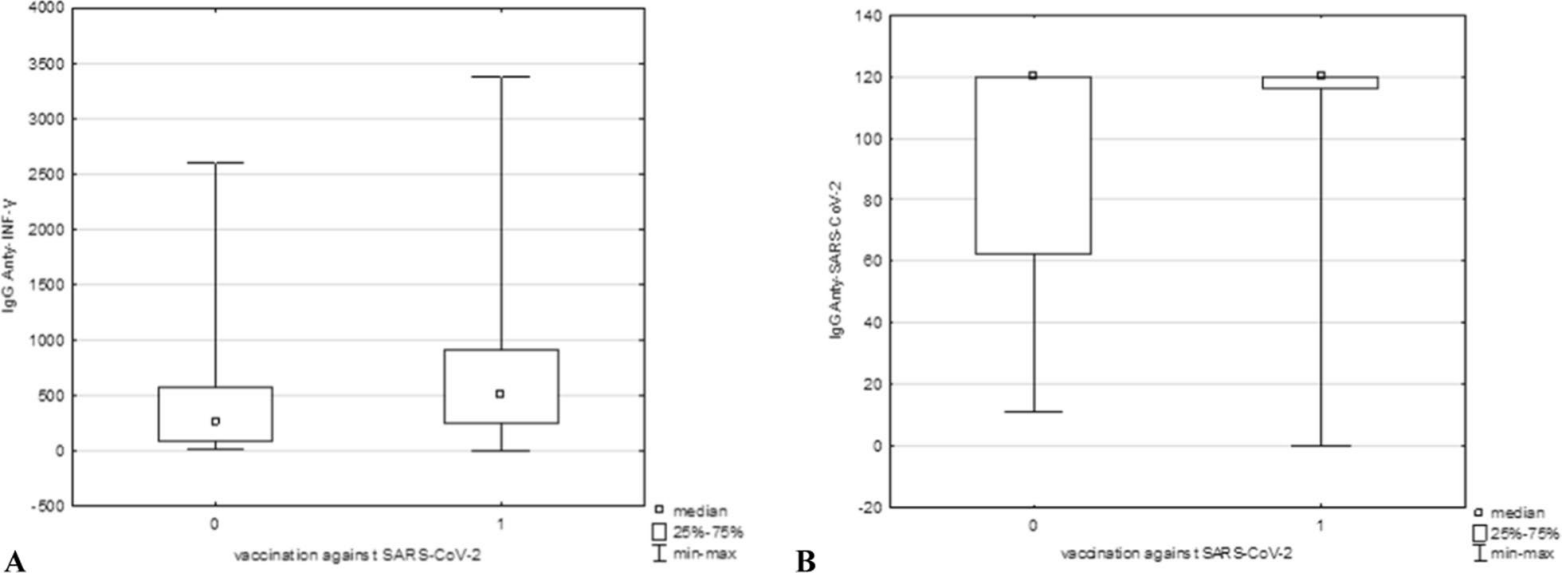
Influence of vaccination against COVID-19 on the level of IgG antibodies specific for INF-γ (**A**) and the SARS-CoV-2 virus (**B**). Anti-INF-γ and anti-SARS-CoV-2 IgG concentrations are given in mlU/ml and RU/ml, respectively

Our results did not reveal a relationship between vaccination and IgG antibody levels against IAV and IBV viruses (p = 0.71415 for IgG anti-IAV and p = 0.11098 for IgG anti-IBV data not shown). However, a statistically significant relationship and a weak correlation were detected between vaccination and the levels of anti-INF-γ and anti-SARS-CoV-2 IgG antibodies (p = 0.00334 and ρ = 0.181343 for anti-INF-γ IgG and p = 0.00057 and ρ = 0.215763 for anti-SARS-CoV-2 IgG) (Fig. 2a, b).

**Fig. 3:**
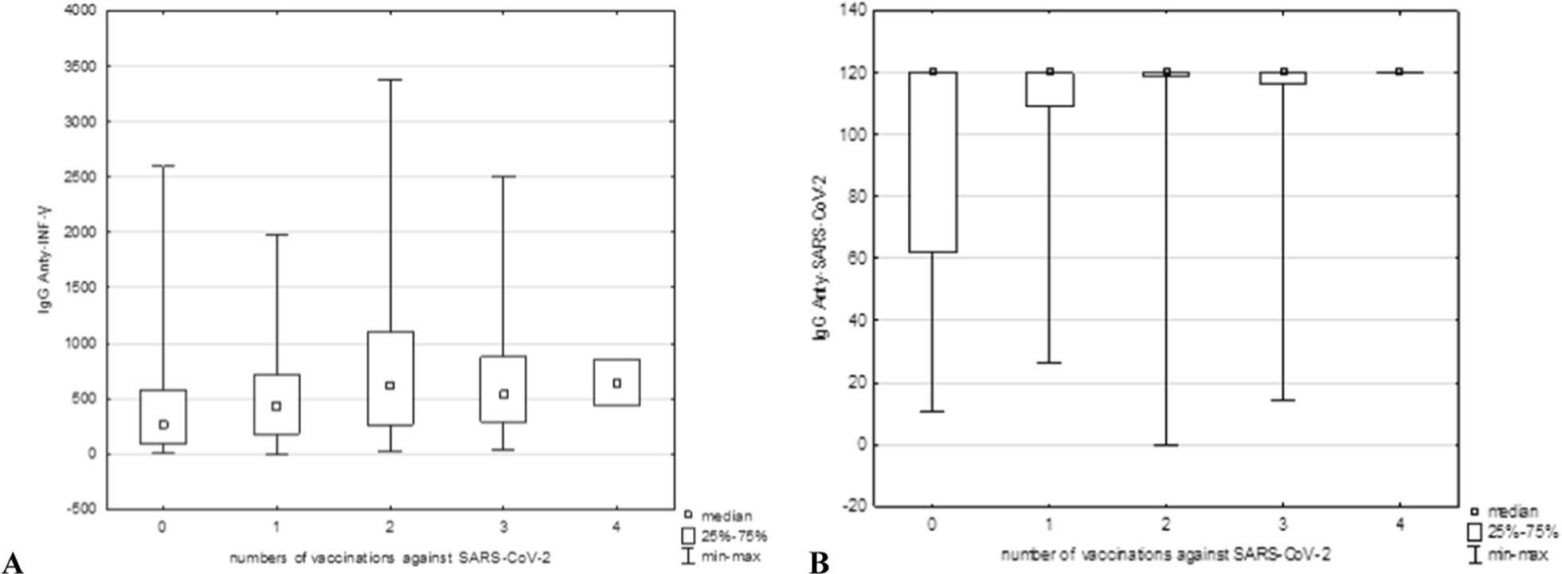
Influence of different number of doses of vaccination against COVID-19 on the level of IgG antibodies specific to INF-γ (**A**) and the SARS-CoV-2 virus (**B**). Anti-INF-γ and anti-SARS-CoV-2 IgG concentrations are given in mlU/ml and RU/ml, respectively

Notably, vaccinations support the immune system, especially by significantly increasing the amount of anti-SARS-CoV-2 IgG antibodies (Fig. 2b). However, analysis via the Kruskal‒Wallis H test did not reveal a significant difference between the level of anti-SARS-CoV-2 IgG and the number of vaccinated individuals (χ^2^(2) = 12.91046; p = 0.0117). Statistically significant relationships were detected only between the level of anti-INF-γ IgG in the unvaccinated group and that after two doses. A weak positive correlation was found for all vaccination doses (ρ = 0.168038; p < 0.05) (Fig. 3a). For anti-SARS-CoV-2 IgG antibodies, relationships between their levels in groups without vaccination and after two doses and between the groups without vaccination and after three doses of vaccines were noted (χ^2^(2) = 19.95816 p = 0.0005). A weak positive correlation was also found among all vaccination doses and anti-SARS-CoV-2 antibodies (ρ = 0.188823; p < 0.05). The higher levels of these antibodies reflected the greater number of patients who received the vaccine (Fig. 3b).

## Discussion

In the Polish climate, we observe the seasonality of respiratory infections. This trend is associated mainly with the flu and the common cold. Defining the etiological agents of upper respiratory tract infections without dedicated tests, such as PCR, is difficult (or even impossible) because of the similarity of symptoms. The viruses responsible for causing the common cold (at least 50% of colds in adults) are mainly rhinoviruses [11]; they cause up to 80% of all respiratory infections in the high season. These viruses usually circulate between September and October, whereas flu viruses have their peak prevalence in the December–March period. This regularity was also reflected in our results, as rhinovirus was the most frequently detected virus in the studied group (n = 33; 8.71%), followed by SARS-CoV-2, with an infection rate of 4.75% (n = 18); swine influenza A H1N1 (2.90%; n = 11); and human coronavirus 229E (2.64%; n = 10) (Table 2). Coronaviruses 229E and OC43 were identified in the 1960s [12] and have since been associated with mild respiratory illnesses; after rhinoviruses, they are the leading cause of common colds [13]. Seasonal coronaviruses do not include SARS-CoV-2 ([14, 15]; https://www.cdc.gov/flu/symptoms/coldflu.htm, accessed on 20 June 2024); however, we detected this virus more often than, e.g., HCoV 229E, which is probably due to its broader circulation in the population since the COVID-19 pandemic. The detection of some cases of swine influenza A H1N1 is connected with the circulation of this subtype in humans. We have decided to take our samples during the fall/winter season, i.e., between October and February, but we want to emphasize that for the present study, we did not specifically select people with ongoing respiratory infections. The volunteers were of random health condition. In most cases, they did not undergo a serious infection at that time.

Healthcare workers and individuals who come into contact with animals (particularly swine and poultry) are two primary occupational groups at increased risk for developing respiratory infections, such as lung infections [16, 17]. In addition, these workers and veterinary personnel are also at increased risk of contracting influenza [18]. Nevertheless, other various environmental factors could contribute to work-related RTIs. For instance, staying in bigger communities, such as schools or military facilities, can frequently lead to outbreaks of human adenovirus [19]. For military personnel, stressors such as intense physical training, energy deficit, or psychological stress can also weaken their immune system and lead to higher RTI morbidity rates [20]. Multiple studies have shown that respiratory tract infections are among the most common in military personnel [21–25]. Accoording to these sources, the most widespread detected viruses are: adenoviruses, SARS-CoV-2, parainfluenza and influenza A and B viruses, metapneumoviruses, rhinoviruses, and RSV in varying order of occurrence.

Bacteria are a less common etiological cause of upper respiratory infections. Nevertheless, a few exceptions can be mentioned, such as bacterial pharyngitis caused by *S. pyogenes* or epiglottitis and laryngotracheitis caused by *H. influenzae* type B [26]. Along with *S. aureus* (n = 70; 18.47%), we found *H. influenzae* (n = 54; 14.24%) and *K. pneumoniae* (n = 37; 9.76%) to be frequently present in our study. These three species are common respiratory pathogens but do not necessarily indicate an infection. The presence of *S. aureus* is not surprising since it is estimated that 70–90% of the general population are at least temporary carriers of this pathogen, and as many as one-third of adults (32%) can be asymptomatic carriers [27]. Other studies have confirmed *S. aureus* nasal colonization [28–31]. According to the mentioned research, soldiers in certain duties-related circumstances might be exposed to increased transmission of *S. aureus* due to physical combat training and limited access to sanitary facilities. Decreased immune system resistance caused by chronic stress or a lack of sleep may also play an important role. *K. pneumoniae* also naturally colonizes human skin, the oral cavity, and the intestines, and it can cause pneumonia [32]. *Haemophilus influenzae* penetrates the organism through the nasopharynx and colonizes this area temporarily or for several months without symptoms (asymptomatic carrier state), but it can also lead to invasive infections. The health condition of the participants greatly influenced the results of our study. The tested population consisted mainly of healthy adults, but 21.90% of them (*n* = 83) reported having cold symptoms in the past two weeks. For those without symptoms, the detected bacteria might have constituted their natural microflora and not an ongoing infection.

Antibody levels produced after exposure to the etiological factor or vaccination are diverse owing to dependencies between age, immune system condition, comorbidities, and the type of agent causing the disease. Generally, the condition of the immune system deteriorates in the elderly population as the lymphocytes responsible for cellular and humoral immunity become defective [33]. Significant immune system impairment resulting in serious complications or increased postinfection mortality occurs in the population over 65 years of age [33]. Our results revealed a weak correlation between age and anti-IAV and anti-IBV IgG levels, both of which increased with age. This phenomenon has been confirmed in other studies [34, 35]. This effect may be associated with repetitive exposure to diverse influenza hemagglutinins (HAs). It has been shown that titers of anti-HA IgG antibodies against pandemic (including H2N2, H3N2, and H1N1) and seasonal flu strains increase in an age-dependent manner [34]. In that research, the studied group was observed within 20 years, and IgG titers and humoral response levels against antigenically stable human cytomegalovirus (HCMV) were measured every five years. Unlike influenza viruses, HCMV remains in the body after the initial infection and enters the latency phase until it is reactivated. Notably, HCMV-specific antibody titers remained at the same level throughout the study period, which may confirm that sequential exposure to antigenically unstable viruses influences the humoral immune response [34].

According to Dietz et al. [36], the levels of anti-SARS-CoV-2 IgG significantly decline only in adults above 75 y.o. Yang et al. [37] reported that although the amounts of antibodies were different in younger people, they showed some trends. They noted that children have higher levels of anti-SARS-CoV-2 IgG than other age groups [37]. This fact may be associated with a milder course of infections in children and differences in innate, adaptive, and heterologous immune functions [38]. A weak but positive correlation between age and the amount of antibodies in adults was also observed [37]. However, in our study, we did not identify any dependence or correlation between age and anti-SARS-CoV-2 IgG levels. This might have been partially determined by the age limits we set for our study group (19–60 years). In addition, due to the relatively short circulation time of the SARS-CoV-2 virus in the environment, there is not enough consistent data concerning the levels of anti-SARS-CoV-2 IgG in different age groups. Additionally, there are many different factors, such as the function of mucosal and systemic innate immunity, vaccines received against unrelated pathogens, endothelium anatomy, and clotting functions, that may influence the severity of COVID-19 and thus IgG levels in different age ranges [38].

As mentioned in the Results, we had too little data to discriminate how past infection or vaccination, separately, influenced the participants' immunity. Having so few people diagnosed with past infections and knowing that other participants might have been sick but undiagnosed or have undergone asymptomatic COVID-19 infection, we analyzed the whole tested population to determine the influence of past infection or vaccination on immunity. The antibody level after vaccination generally reflects the degree of protection against future diseases. Our research aimed to assess the effectiveness of two ways of building immunity against flu and COVID-19, i.e., receiving vaccinations and contracting pathogens. Immunity is influenced by various factors, among which the agent's character is crucial. Pappas [11] indicated that “colds are common because some of the causative viruses do not produce lasting immunity after infection, and some viruses have numerous serotypes.” In general, we can assume that two types of viruses cause colds. Some of them, such as RSV, PIV, and HCoV, can infect a person multiple times with the same serotype without allowing their body to build up long-term immunity after the initial infection [39]. For this reason, subsequent doses of the vaccines are recommended to keep the person immune. Another type of cold viruse is one with numerous serotypes, each capable of infecting an individual only once. These viruses include RV, AdV, EV, and influenza viruses [39], and they can lead to durable immunity following infection, though it is limited to a specific serotype. As reinfection with the same serotype is uncommon, annual vaccination updates may be necessary to ensure immunity against the current circulating serotypes.

In the case of influenza, annual vaccinations may increase antibody levels and thus better protect people against severe infections than those who receive only one dose of the vaccine [40]. Surprisingly, our studies revealed no relationship or correlation between the number of vaccinated individuals in the last 12 months and the levels of anti-IAV and anti-IBV IgG (data not shown). The antibody levels in the vaccinated and unvaccinated groups remained similar. Nevertheless, the explanation for this phenomenon may be the limitation of our research: the relatively small size of the study population and the fact that only vaccinations from the last 12 months were declared. In addition, the percentages of people vaccinated (9.5%; 36/379) and diagnosed with influenza (4.4%; 17/379) during the previous season were too small to influence the overall effect. In people who did not declare vaccination during the previous season, IgG antibodies against influenza A and B could have accumulated from vaccinations or infections in earlier years, affecting IgG levels. Interestingly, vaccine effectiveness, as measured by antibody levels, does not always correlate with real clinical protection from laboratory-confirmed influenza. The mechanisms of cellular immune and T lymphocyte functions may play important roles in protection against infections [40]. As shown above, the mechanism of the immune reaction in SARS-CoV-2 infection has a different course. Antibodies against the SARS-CoV-2 virus remain at a high level for only approximately 3‒6 months; in some cases, they are even shorter, and then their amount decreases dramatically [41–43]. Vaccination efficiently increases both humoral and cellular responses to SARS-CoV-2 [44], which, in our study, was measured by evaluating anti-SARS-CoV-2 and anti-INF-γ IgG levels, respectively. These levels, especially the humoral response (Fig. 2b), were notably greater in the vaccinated population than in the unvaccinated group, which is consistent with earlier research. Moreover, the intake of each subsequent vaccine dose strongly correlated with higher levels of specific antibodies (Fig. 3b), suggesting that booster vaccination may contribute to prolonged immunity against severe COVID-19 infections.

### Limitations

The project designs limited the size of the study group. There was no information about when the participants received the COVID-19 vaccine.

## Conclusions

In our research, we assessed the prevalence of 34 respiratory pathogens in soldiers and civilian military employees aged 19–60 years. The study group consisted mainly of healthy people; cold symptoms within the previous two weeks were declared by almost 22% of the participants. At least one respiratory pathogen was detected in 78% of the tested people, and nearly one-third were coinfected with two or more pathogens. The three most abundant bacteria we detected were *S. aureus*, *K. pneumoniae,* and *H. influenzae,* and the most common virus was rhinovirus. To evaluate the immunity of the tested participants with respect to flu and COVID-19, we measured the levels of their specific antibodies and observed the dependence and weak correlation between vaccination and anti-SARS-CoV-2 IgG levels. The results also revealed a greatly increased amount of these antibodies after consecutive vaccine doses, confirming the legitimacy of their use. For the anti-influenza antibodies, we observed a dependence between their levels and the age of the participants with increasing titers in the older participants. The results of our research may contribute to increasing awareness of the need to monitor health, especially in the field of respiratory diseases, as well as the validity of vaccinations. It will also allow for assessing the effectiveness of intervention activities to prevent epidemic events.

## Data Availability

The raw data obtained during this study are available on request from the corresponding author.

## Abbreviations

ARI: Acute respiratory illness
ELISA: Enzyme-linked immunosorbent assay
EV: Enterovirus
FTD: Fast Track Diagnostics®
HAdV: Human adenovirus
HA: Influenza hemagglutinin
HBoV: Human bocavirus
HCMV: Human cytomegalovirus
HCoV 229E: Human coronavirus 229E
HCoV NL63: Human coronavirus NL63
HCoV OC43: Human coronavirus OC43
HMPV A&B: Human metapneumovirus A and B
HPIV03: Human parainfluenza virus 3
HRV: Human rhinovirus
IAV: Influenza A virus
IAV(H1N1)swl: Influenza A (H1N1) virus (swine lineage)
IBV: Influenza B virus
ICU: Intensive Care Unit
ICV: Influenza C virus
IE: Internal control
IFN-γ: Interferon-gamma
IGRA: Interferon-Gamma Release Assay
IVD: In vitro diagnostics
LRTI: Lower respiratory tract infection
NIPH-NIH: National Institute of Public Health-National Institute of Hygiene
PCR: Polymerase chain reaction
PIV: Parainfluenza virus
RSV: Human respiratory syncytial virus
RTI: Respiratory tract infection
RT-PCR: Reverse-transcriptase polymerase chain reaction
URI: Upper respiratory infection
